# Evaluating the long-term effectiveness of a structured telehealth obesity program in children and adolescents: A retrospective matched-control study

**DOI:** 10.1016/j.obpill.2025.100206

**Published:** 2025-08-29

**Authors:** Nora Struckmeyer, Torben Biester, Chantal Weiner, Evelin Sadeghian, Cathrin Guntermann, Laura Galuschka, Kisa von Stuelpnagel, Jantje Weiskorn, Kerstin Kapitzke, Karin Lange, Thomas Danne, Rebecca Toenne, Felix Reschke

**Affiliations:** a Children's Hospital AUF DER BULT, Hanover, Germany; b GynPraxis Hannover, Hannover, Germany; c Department of Sports Medicine, Hannover Medical School, Hannover, Germany; d Medical Psychology, Hanover Medical School, Hanover, Germany; e Breakthrough T1D, New York, USA; f Betreuungsnetz, Netzwerk zur Versorgung schwerkranker Kinder e.V., Germany

**Keywords:** Adolescent obesity, Dietary behavior, Health-related quality of life, Pediatric obesity, Telehealth intervention, Weight management

## Abstract

**Background:**

Childhood obesity is a growing global health crisis, driven by poor diet, reduced physical activity, and psychosocial distress. The COVID-19 pandemic amplified these factors, contributing to rising BMI and impaired health-related quality of life (HrQoL). Telehealth offers a promising, scalable modality to deliver multimodal obesity care. This study evaluated the long-term effectiveness of a structured pediatric telehealth intervention compared to historical in-person treatment.

**Methods:**

This retrospective cohort study analyzed data from 237 children and adolescents with obesity treated at a single academic center. Between 2020 and 2022, 117 participants received a 12-month structured lifestyle intervention via telehealth. A historical cohort (n = 120; 2017–2019) received the same intervention in person. Clinical outcomes were assessed at baseline and after 12 months; the telehealth group was additionally followed up at 24 and 36 months. Primary outcome was change in BMI standard deviation score (BMI SDS). Secondary outcomes included physical fitness (6-min walk test), insulin resistance (HOMA index), lipid profile, dietary behavior (K-FFL), eating regulation (K-FEV), and HrQoL (KINDL-R).

**Results:**

Both groups achieved significant reductions in BMI SDS after 12 months, with sustained improvements in the telehealth group through 36 months (Δ = −0.18; p < 0.05). Physical performance and HOMA index improved in both cohorts. Telehealth participants showed greater improvements in healthy dietary behavior, cognitive appetite regulation, and Health-related quality of life HrQoL, especially in emotional and family domains. No adverse events occurred; adherence exceeded 85 %.

**Conclusion:**

A structured telehealth lifestyle intervention is safe, effective, and sustainable for pediatric obesity management. These findings support telehealth as a clinically viable and sustainable model for pediatric obesity care, recognizing that both weight reduction and weight stabilization may contribute to improved long-term outcomes.

## Graphical abstract

**Figure:**
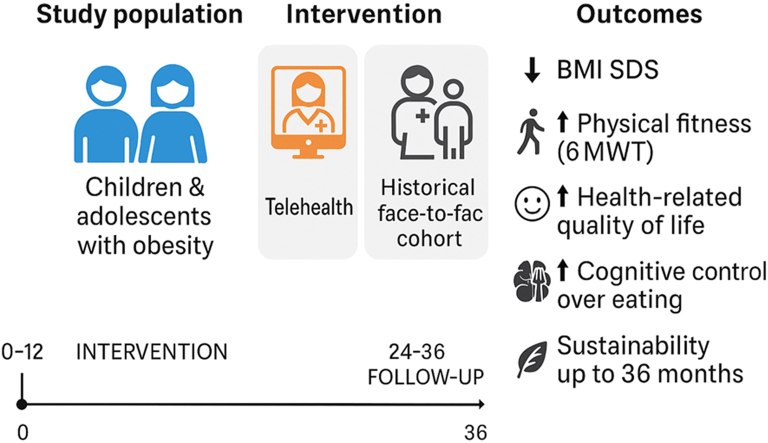

## 1 Introduction

Childhood and adolescent obesity has emerged as a critical global health concern with escalating prevalence over the past decades. According to the World Health Organization (WHO), the number of children and adolescents aged 5–19 years living with overweight or obesity has increased more than tenfold since 1975, affecting over 340 million individuals worldwide in 2016 [1]. Pediatric obesity is associated not only with somatic comorbidities such as type 2 diabetes, dyslipidemia, hypertension, and musculoskeletal complications, but also with substantial psychosocial sequelae including depression, low self-esteem, social stigmatization, and diminished health-related quality of life (HrQoL) [[2], [3], [4], [5]].

Maladaptive lifestyle behaviors remain central contributors to obesity development. High consumption of energy-dense, nutrient-poor foods (e.g., sugar-sweetened beverages, processed snacks, fast food), low intake of fiber-rich foods (vegetables, fruits, whole grains), insufficient physical activity, and increasing sedentary time—particularly screen exposure—promote positive energy balance and adiposity accumulation [[6], [7], [8], [9]]. In addition, genetic, epigenetic, and biological factors substantially influence obesity risk. Twin and family studies estimate that 40–70 % of BMI variability is heritable, and dysregulation of appetite control, energy expenditure, and neuroendocrine signaling (e.g., leptin-melanocortin axis dysfunction) further predisposes youth to early-onset obesity [10]. Environmental and behavioral factors may amplify these biological vulnerabilities, highlighting the multifactorial nature of pediatric obesity.

These risk factors were exacerbated during the COVID-19 pandemic, which disrupted daily routines, schooling, physical activity, and family dynamics. Evidence indicates pandemic-associated increases in unhealthy food consumption, reduced intake of fresh produce, lower physical activity, and deterioration in mental well-being among children and adolescents [[11], [12], [13]].

Given these challenges, scalable interventions that deliver evidence-based obesity treatment independent of in-person constraints are urgently needed. Comprehensive, multimodal programs—including nutritional education, structured physical activity, behavioral therapy, and medical monitoring—remain the cornerstone of pediatric obesity management but are often limited by logistical, geographic, and socioeconomic barriers [[14], [15], [16], [17], [18]]. Telehealth offers a promising solution to expand access, improve adherence, and maintain intervention fidelity, with rapid adoption during the COVID-19 pandemic providing an unprecedented opportunity to evaluate long-term effectiveness [19,20].

This study investigates the effectiveness and sustainability of a structured, multimodal pediatric obesity intervention delivered via telehealth compared to a historical face-to-face cohort receiving the same program. The 12-month intervention was followed by a 24-month observational follow-up, assessing BMI SDS, metabolic parameters, physical fitness, dietary behavior, appetite regulation, and HrQoL. We hypothesize that telehealth delivery achieves outcomes equivalent to or better than conventional face-to-face programs and offers a durable, patient-centered approach to obesity care in youth.

## 2 Methods

### 2.1 Study design

**Fig. 1:**
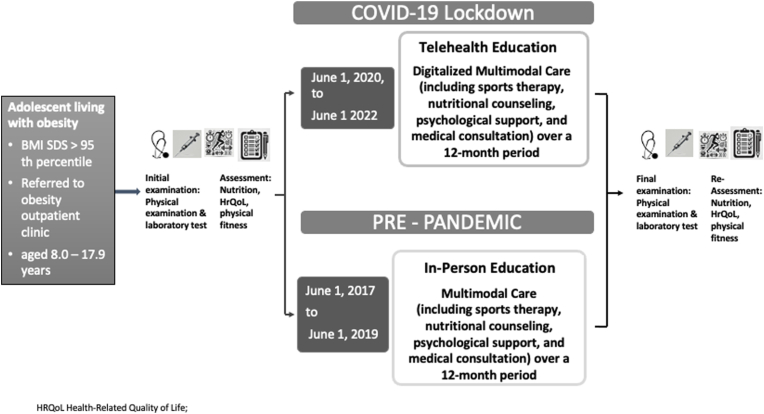
Flowchart illustrating the structure and timeline of the multimodal obesity intervention delivered either via telehealth (during the COVID-19 pandemic; June 2020–June 2022) or in-person (pre-pandemic; June 2017–June 2019). Both cohorts received identical 12-month interventions including sports therapy, nutritional counseling, psychological support, and medical consultation. Clinical assessments (anthropometry, laboratory parameters, physical fitness) and self-report questionnaires (dietary behavior, HrQoL) were conducted at baseline and after 12 months. Only the telehealth group underwent extended follow-up with reassessments at 24 and 36 months. *Abbreviations: BMI SDS, Body Mass Index Standard Deviation Score; HrQoL, Health-related Quality of Life*.

This retrospective observational cohort study assessed the long-term effectiveness of a structured, multidisciplinary pediatric obesity intervention delivered via telehealth. A historical cohort that received the identical program via in-person sessions prior to the COVID-19 pandemic served as the control group. The intervention period spanned 12 months and was followed by a 24-month observational follow-up, totaling 36 months. Due to pandemic-related constraints, long-term follow-up data were available only for the telehealth cohort; the historical face-to-face cohort was evaluated solely during the intervention phase. Participants in the telehealth group were enrolled between June 2020 and June 2022, while the face-to-face cohort participated between June 2017 and June 2019 (Fig. 1).

This analysis utilized routinely collected clinical data from the real-world KiCK program (Kinder und Jugend intensiv Coaching im Krankenhaus) [14]. All procedures adhered to standardized institutional protocols, consistent across both cohorts. The study complied with the Declaration of Helsinki and was reviewed by the Ethics Committee of Hannover Medical School (MHH), which confirmed that no formal vote was required due to its retrospective, non-interventional nature.

### 2.2 Setting and participants

The study was conducted at the Children's Hospital AUF DER BULT in Hanover, Germany, involving a single-center design. Eligible participants were children and adolescents aged 8.0–17.9 years with obesity, defined as a BMI standard deviation score (BMI SDS) above the 97th percentile, according to German reference data (18) Referrals were made by primary care physicians and pediatricians.

Exclusion criteria included:

• Presence of chronic somatic, psychiatric, or acute psychiatric diseases (e.g., endocrine disorders, genetic syndromes)
• Inability to participate in digital sessions (telehealth group only) or attend group-based sessions

Participants were enrolled consecutively and matched across groups using propensity score matching (PSM).

### 2.3 Intervention: KiCK program

All participants received the KiCK intervention (Kinder-und Jugendintensiv-Coaching im Krankenhaus), a standardized, 12-month outpatient obesity management program certified by the German Obesity Society (DAG). The program follows a multimodal, family-based approach and is delivered by an interdisciplinary team consisting of pediatricians, psychologists, dietitians, and exercise scientists, all certified in pediatric obesity care.

The intervention combines four core components:

1. **Medical supervision:** Regular pediatric assessments (baseline, 12 months), anthropometric measurements, metabolic blood testing, and physical examinations.
2. **Nutritional counseling:** Delivered weekly or biweekly, focusing on dietary literacy, portion control, and behavior change based on national nutrition guidelines.
3. **Exercise therapy:** Weekly live sessions (90–120 min) with home-based endurance and strength activities, complemented by physical performance testing (6MWT) at standardized intervals.
4. **Psychological and behavioral therapy:** Structured modules on stress regulation, emotion management, motivation, and family dynamics, provided via group and individual sessions.

The face-to-face cohort attended sessions in person in a hospital-based setting, while the telehealth cohort received the same curriculum via secure video conferencing (Cisco Webex, San Jose, Ca, USA). Live online sessions occurred once to twice weekly, with additional digital content ("KICK of the Week") sent via email. Parents and family members were actively included in educational and physical activity modules. All participants received approximately 180–360 min of structured therapy per week. Regular participation (≥80 % of sessions) was defined as adherent.

The program structure, therapeutic content, and delivery frequency were kept consistent across cohorts, ensuring comparability between telehealth and in-person modalities. The curriculum and quality standards correspond to national pediatric obesity treatment guidelines (S2/S3, AGA/DAG).

A detailed description of the KiCK program structure and certification has been published previously [14].

### 2.4 Outcome measures

Clinical and laboratory outcomes were assessed at baseline and at 12 months, representing the intervention period. These included anthropometric measurements, blood pressure, lipid profile, glycemic indices (fasting glucose, insulin, HOMA-IR), liver enzymes, thyroid function (TSH), and renal function (creatinine). Anthropometry was performed using calibrated instruments, and venous blood samples were analyzed in certified laboratories. HOMA-IR was calculated using the formula: fasting insulin (μU/mL) × fasting glucose (mg/dL)/405.

At 24 and 36 months, follow-up assessments were restricted to BMI SDS (self-measured at home by participants or parents following standardized instructions), physical performance via the 6-min walk test (6MWT), and self-reported outcomes assessed through validated questionnaires. These included:

• Health-related quality of life via the KINDL-R (Kinder Lebensqualitätsfragebogen), which evaluates physical and emotional well-being, self-esteem, and family and peer relationships [21].
• Dietary intake was assessed using the validated Kinder-Food Frequency List (K-FFL), a standardized semi-quantitative instrument designed to capture habitual consumption frequencies of 37 food items across eight food groups: cereal products/potatoes, vegetables/salad, fruits, dairy products, meat/fish products, fats, beverages, and sweets/fast food. Participants reported consumption using predefined frequency categories: “3–5 servings per day,” “1–2 servings per day,” “4–6 servings per week,” “1–3 servings per week,” and “rarely or never.”. For analytical purposes, dietary patterns were categorized as either “healthy” or “unhealthy” according to previously established classification frameworks [13]. For nutrient-dense food items (e.g., fruits, vegetables, whole grains, dairy), intake frequencies of ≥4 servings per week (i.e., “3–5/day,” “1–2/day,” or “4–6/week”) were classified as healthy. Lower consumption frequencies were considered unhealthy. Conversely, for energy-dense or nutrient-poor items (e.g., sugar-sweetened beverages, sweets, fast food), the same threshold (≥4 servings/week) was categorized as unhealthy [22].
• The analysis focused on selected obesity-relevant food categories [14].
• Eating behavior via the K-FEV (Kinder-Essverhaltenfragebogen), assessing cognitive restraint, disinhibition, and hunger perception. This instrument demonstrates strong psychometric properties and is widely applied in pediatric obesity research [23].
• Survey data at 24- and 36-month follow-up were collected through a secure, web-based platform (SurveyMonkey, SurveyMonkey Europe UC, Ireland), ensuring standardized administration and accessibility.

### 2.5 Primary outcomes

• BMI SDS
• Health-related quality of life (KINDL-R)

### 2.6 Secondary outcomes

• Physical fitness (6MWT)
• Metabolic parameters (fasting glucose, insulin, HOMA-IR, HbA1c, lipid profile, TSH, gamma-GT, creatinine)
• Dietary behavior (K-FFL)
• Eating behavior (K-FEV)

The KiCK telehealth intervention has previously shown short-term effectiveness in improving lifestyle behavior and metabolic health during the COVID-19 pandemic [14]. This study expands on prior findings by incorporating a long-term perspective on sustainability and efficacy.

### 2.7 Statistical analysis

Statistical analyses were conducted using SPSS Version 29.0.2.0 (20), IBM, USA, and Excel, Microsoft 365, USA. This secondary analysis compared two clinical cohorts using 1:1 propensity score matching (nearest-neighbor without replacement) based on age, sex, BMI SDS, lipid profile, and HOMA-IR to reduce baseline confounding. Behavioral and psychosocial parameters (e.g., dietary intake, physical activity, health-related quality of life) were not included in the matching model due to contextual differences related to the COVID-19 pandemic and to avoid overfitting. Between-group differences were tested using independent t-tests or Mann–Whitney U tests for continuous variables and chi-square tests for categorical variables. Dietary behavior variables, derived from the K-FFL, were treated as categorical proportions (e.g., % of participants with healthy intake frequency per food item) and analyzed using chi-square tests accordingly. To account for repeated measurements across multiple time points (baseline, 12 months, 24 months, 36 months), mixed-effects models for repeated measures (MMRM) were applied. Covariance structure assumptions (e.g., compound symmetry) were tested using Mauchly's test; where violated, alternative structures such as unstructured or autoregressive [AR(1)] were used. Between-group effects were prioritized in all longitudinal models. Within-group comparisons over time were reported descriptively or as exploratory analyses.

Missing data were addressed using multiple imputation via predictive mean matching, applied separately by group and time point. Subgroup analyses by sex were conducted post hoc."

Effect sizes and 95 % confidence intervals (CIs) were reported for key outcomes to estimate clinical relevance. Descriptive statistics are presented as means ± standard deviation (SD), medians with interquartile ranges (IQR), or percentages, as appropriate. All statistical tests were two-sided, with a significance threshold of p < 0.05.

No power calculation was conducted due to the retrospective, real-world cohort design. The analysis was not subject to oversight by a Data Safety Monitoring Board or adjudication committee.

## 3 Results

### 3.1 Participant demographics and baseline characteristics

Baseline demographic and clinical characteristics of the full study population are presented in Table 1. The historical face-to-face cohort (June 2017–June 2019) included 120 participants (51.7 % female, 48.3 % male; mean age: 12.1 ± 4.0 years), while the telehealth cohort (May 2020–May 2022) comprised 117 participants (52.1 % female, 47.9 % male; mean age: 12.9 ± 4.1 years).

**Table 1:** Baseline characteristics of participants in the face-to-face and telehealth intervention groups following propensity score matching.

	Matched Data from 2017 to 2019 (PRE-COVID)			Data from May 2020–May 2022 (COVID)			Reference range	Propensity Score Matching p Value
**Sex**	♀	♂	total	♀	♂	total		
**N (%)**	62 (51,7)	58 (48,3)	120	61 (52.1)	56 (47.9)	117		
**Age (years) [Mean** ± **SD]**	11.7 ± 4.1	12.8 ± 3.7	12.1 ± 4.0	12.3 ± 4.7	13.7 ± 4.2	12.9 ± 4.1		0.825
**Height (cm) [Mean** ±**SD]**	152.9 ± 11.4	163.4 ± 16.5		154.9 ± 12.0	161.5 ± 17.9			
**Weight (kg) [Mean** ±**SD]**	74.3 ± **11.9**	84.3 ± **27.1**		72.2 (± **15.7)**	81.8 ± **25.6**			
**Weight z score**	2.19 ± **0.27**	2.31 ± **0.33**	2.27 ± **0.35**	2.11 ± **0.31**	2.24 ± **0.29**	2.18 ± **0.30**		1.000
**BMI (kg/m2)**	29.8 ± **3.7**	32.8 ± 4.1		31.5 ± **3.2**	30.2 ± **4.1**			
**BMI z score**	2.21 ± **0.39**	±**2.27** ± **0.29**	2.24 ± **0.36**	2.31 ± **0.41**	2.47 ± **0.41**	2.41 ± **0.38**		0.825
**RR SDS (systolic)**	0.69	**0.81**	0.7 (0.4; 1.2)	0.39	**0.71**	0.5 (0.4; 1.1)		0.825
**RR SDS (diastolic)**	0.42	**0.53**	0.5 (0.2; 0.6)	0.32	**0.53**	0.4 (0.2; 0.6)		1.000
**Cholesterol (mmol/L)**	5.27	**5.03**	5.13 (4.34; 5.52)	5.17	**5.33**	5.23 (4.12; 5.63)	<4.40	1.000
**LDL cholesterol (mmol/L)**	2.85	**3.03**	2.88 (2.35; 3.12)	2.75	**3.13**	2.88 (2.49; 3.22)	<2.85	0.825
**HDL cholesterol (mmol/L)**	1.38	**1.57**	1.47 (1.06; 1.57)	1.18	**1.51**	1.41 (1.15; 1.61)	>1.55	1.000
**Triglycerides (mmol/L)**	1.77	**1.89**	1.91 (1.23; 1.99)	1.75	**1.87**	1.79 (1.22; 2.20)	<1.80	1.000
**HOMA index**	4.0	**3.7**	3.8 (3.2; 5.6)	4.0	**3.7**	3.8 (3.2; 5.6)	<2.0	1.000
**6 min walking test (meters)**	598	**622**	615 (573; 644)	598	**622**	602 (563; 618)		0.700

Baseline demographic, anthropometric, metabolic, cardiovascular, hepatic, renal, and physical performance characteristics of the face-to-face and telehealth intervention groups following propensity score matching.

No statistically significant differences were observed between the two groups at baseline in key anthropometric variables (BMI SDS, weight, height), cardiovascular parameters (blood pressure), metabolic indices (fasting glucose, insulin, HOMA-IR, lipid profile), liver function (GOT), renal function (creatinine), or physical performance (6-min walking test). (Table 1).

### 3.2 Clinical and laboratory parameters after 12-month intervention

*BMI SDS*: Both groups demonstrated a reduction in BMI SDS after 12 months. The telehealth group showed a mean decrease of Δ = −0.18 (p < 0.05), comparable to the reduction observed in the face-to-face group, indicating the efficacy of both delivery modes in improving weight status (Table 1). Between-group comparisons after 12 months showed comparable improvements in key outcomes, while the telehealth cohort exhibited additional gains in dietary and psychosocial domains.

**Fig. 2:**
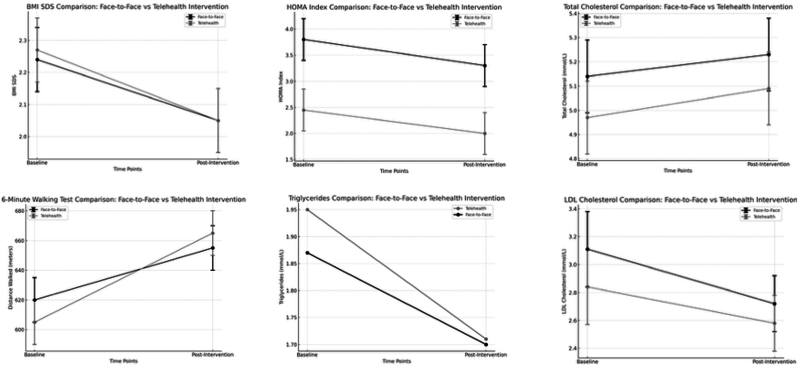
*„*Changes in anthropometric, metabolic, and physical fitness parameters in adolescents with obesity after 12-month multimodal intervention, comparing face-to-face (pre-pandemic) and telehealth (during pandemic) delivery formats. Parameters include BMI SDS, HOMA index, total and LDL cholesterol, triglycerides, and 6-min walking distance.-„

*Physical Fitness (*6MWT*)*: The 6-min walking test distance increased significantly in both cohorts. Participants in the telehealth group improved by 97.0 m (p < 0.05), suggesting enhanced aerobic capacity (Fig. 2).

*Insulin Resistance (HOMA-IR)*: The telehealth group exhibited a significant reduction in HOMA-IR (Δ = −1.4, p < 0.05), reflecting improved insulin sensitivity. A similar trend was observed in the face-to-face group.

*Metabolic Markers*: Total cholesterol and LDL cholesterol decreased by 0.2 mmol/L in the telehealth group, aligning with changes in the face-to-face group. Triglycerides showed similar trends. No significant alterations were detected in GOT or HbA1c levels.

Between-group comparisons after 12 months revealed statistically similar improvements in BMI SDS and physical fitness, with the telehealth cohort showing additional benefits in dietary behavior and HrQoL.

### 3.3 Dietary behavior (K-FFL)

Baseline dietary behavior, assessed using the K-FFL questionnaire, is summarized in Table 2. At the start of the program, participants in the telehealth group already showed more favorable consumption patterns in several domains: :

• **Sugar-sweetened beverages:** After 12 months, the telehealth group demonstrated a significant reduction in sugar-sweetened beverage intake compared to baseline. The proportion of participants with a high (unhealthy) consumption frequency decreased to 65.5 % (p < 0.001), indicating a measurable shift toward healthier beverage habits. This pattern was comparable to the face-to-face group at follow-up.
• **Fast food**: Healthy consumption frequency was observed in only 4.7 % of the telehealth group compared to 31.2 % in the face-to-face group (p < 0.01), suggesting a less favorable outcome in this specific domainA significantly higher proportion of participants in the telehealth group reported healthy vegetable intake compared to the face-to-face group (89.5 % vs. 36.5 %; p < 0.01), based on categorical classification derived from the K-FFL
• **Fruit, cheese, and wholegrain intake**: The telehealth cohort demonstrated higher rates of healthy consumption patterns for these food categories (p < 0.05 for all comparisons).

**Table 2:** Baseline dietary behavior characteristics of participants in the telehealth and face-to-face cohorts, assessed using the Kinder-Food Frequency List (K-FFL).

Food content	Historical Face to Face (Jun 2017–Jun 2019) N = 120		Telehealth Intervention (Jun 2020–2022) N = 117		Comparison between Face-to-Face and Telehealth	
	Healthy consumption frequency % (95 % CI)	Unhealthy consumption frequency % (95 % CI)	Healthy consumption frequency % (95 % CI)	Unhealthy consumption frequency % (95 % CI)	p∗ (Healthy consumption)	p∗ (Unhealthy consumption)
Soft drinks	50.3 (34.9–66.7)	49.7 (31.3–64.1)	34.5 (20.5–44.7)	65.5 (51.3–87.5)	<0.05	<0.001
Mineral water, tea, water	69.2 (51.8–80.2)	30.8 (19.2–49.8)	50.4 (37.0–67.8)	49.6 (28.2–61.2)	<0.05	<0.001
Milk, Cocoa	55.9 (40.1–71.2)	44.1 (26.3–57.7)	48.4 (30.6–55.3)	51.6 (41.8–60.0)	Ns	P < 0.05
Yoghurt, buttermilk, curd	48.5 (34.1–59.8)	51.5 (34.3–61.2)	50.3 (37.9–66.7)	49.7 (38.0–59.8)	ns	ns
Cheese	45.3 (31.4–57.8)	54.7 (37.9–72.5)	59.8 (48.5–72.2)	40.2 (31.2–57.8)	<0.05	<0.05
Meat/suasages	27.8 (11.2–45.3)	72.2 (56.4–87.8)	12.8 (2.5–31.2)	87.2 (62.9–95.4)	<0.05	<0.01
Fish	8.7 (1.9–16.5)	91.3 (84.8–99.9)	3.7 (0.4–8.2)	96.3 (82.8–99.9)	<0.05	ns
Mixed bar, white bread, rolls	47.4 (33.6–61.2)	52.6 (33.1–63.7)	58.4 (41.1–70.7)	41.6 (37.9–66.0)	ns	ns
Wholemeal bread, wholemeal rolls	27.8 (14.9–44.7)	72.2 (57.9–82.1)	23.5 (11.7–45.2)	76.5 (57.2–82.1)	ns	ns
Rice/Pasta	28.7 (14.7–42.6)	72.3 (56.2–88.2)	10.7 (2.8–22.1)	89.3 (76.7–98.6)	ns	<0.05
Fresh fruit	37.9 (19.9–44.3)	62.1 (45.0–76.3)	62.9 (45.1–78.5)	37.1 (13.8–47.9))	<0.05	<0.05
Vegetables/fresh/frozen)	36.5 (23.1–51.4)	63.5 (44.2–71.9)	10.5 (2.8–20.1)	89.5 (77.6–98.4)	<0.05	<0.01
Fastfood/Delivery Food	31.2 (24.6–43.2)	68.8 (58.3–77.8)	4.7 (0.1–10.9)	95.3 (87.8–99.9)	<0.05	<0.01
Chocolate/Sweets	22.9 (11.6–30.2)	77.1 (51.2–85.4)	7.8 (3.2–16.4)	92.2 (69.7–98.9)	<0.05	<0.001
Chips, salty nuts, snack biscuits	28.9 (20.7–39.8)	71.1 (59.9–78.5)	4.2 (0.0–14.2)	95.8 (87.3–99.9)	<0.05	<0.01
Gummi bears, wine gum	37.8 (24.3–55.1)	62.2 (47.2–73.0)	13.8 (5.4–17.1)	86.2 (78.0–92.1)	<0.05	<0.01
Cakes, tartes, biscuits	54.7 (39.8–70.1)	45.3 (42.6–67-1)	78.9 (66.1–88.4)	21.1 (8.7–34.8)	<0.05	<0.001

Caption: Dietary patterns were assessed using the validated Kinder-Food Frequency List (K-FFL) and categorized into "healthy" and "unhealthy" frequency of consumption according to established criteria (see Methods). Data reflect group proportions reporting healthy dietary behaviors at baseline for key obesity-related food items.

**Sweets and snack foods**: The proportion of participants with unhealthy intake of chips, sweets, and gummy bears was significantly lower in the telehealth group (p < 0.01 to p < 0.001).While most participants demonstrated increased intake of fruits and vegetables, only a minority met the international recommendation of ≥5 servings/day.

### 3.4 Health-related quality of life (KINDL-R)

**Fig. 3:**
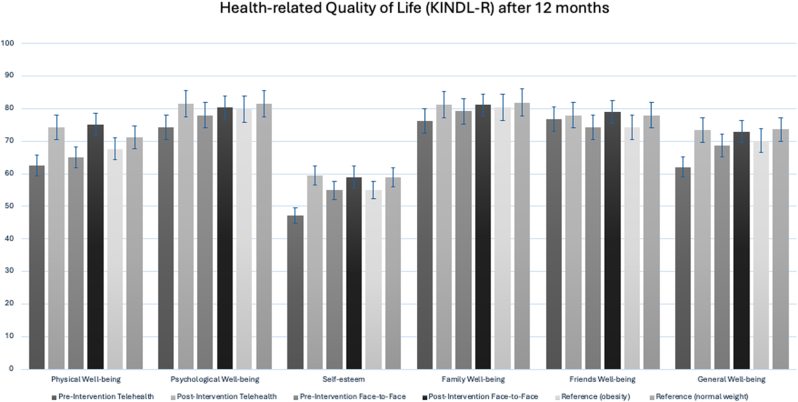
Health-related quality of life (HRQoL) in adolescents with obesity before and after a 12-month multimodal intervention, assessed using the KINDL-R questionnaire. Results are shown for telehealth and face-to-face intervention groups, compared to reference values for peers with obesity and normal weight.

Self-reported HrQoL scores improved significantly in the telehealth cohort. Total KINDL-R scores increased from 62.1 at baseline to 73.4 after 12 months (p < 0.001), with continued gains at 24 (74.1) and 36 months (75.1), particularly in emotional well-being and family-related domains (Fig. 3).

### 3.5 Eating behavior (K-FEV)

**Fig. 4:**
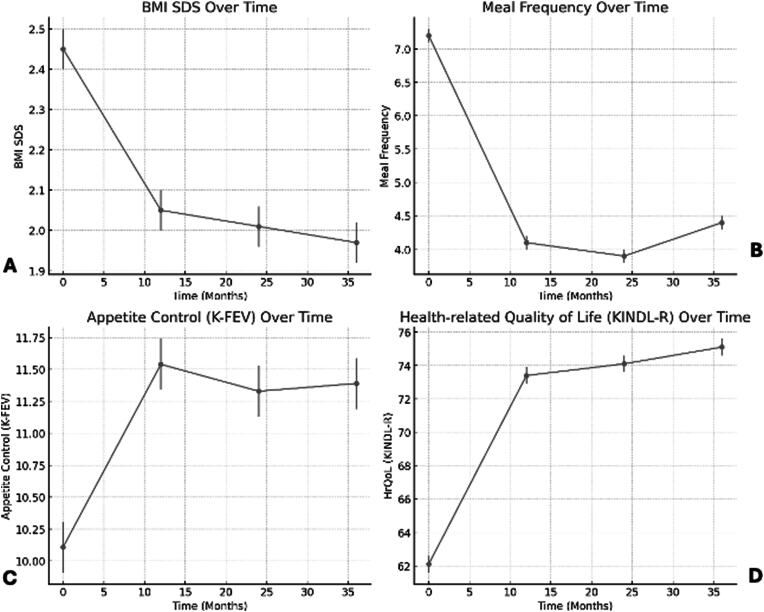
Longitudinal outcomes over 36 months in adolescents with obesity undergoing multimodal intervention. (A) Reduction in BMI SDS; (B) Decrease and partial normalization of meal frequency; (C) Improvement in appetite control (K-FEV); (D) Increase in health-related quality of life (HRQoL, KINDL-R).

Cognitive control over eating, as measured by the K-FEV, improved significantly in the telehealth group: baseline score 10.11 increased to 11.54 at 12 months (p < 0.01), with sustained values at 24 months (11.33) and 36 months (11.39). The face-to-face group also showed improvement, though less pronounced (10.02–10.62; p < 0.05; Fig. 4).

### 3.6 Sustainability of effects at follow-up

**Fig. 5:**
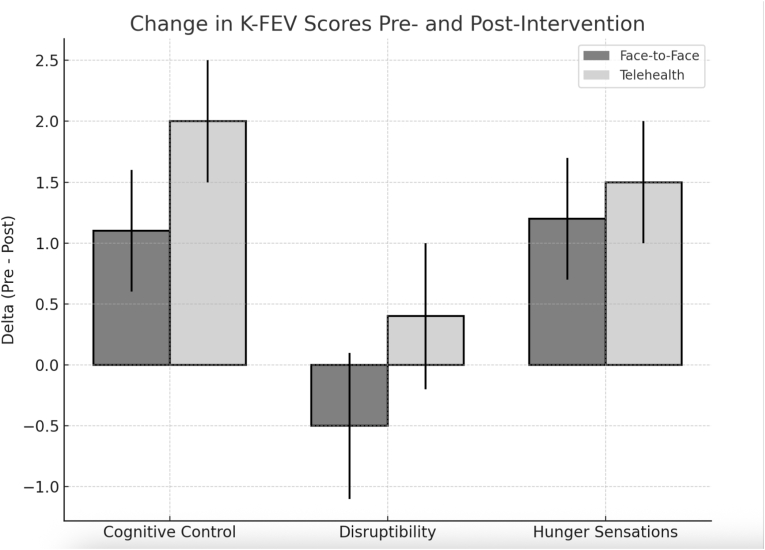
Changes in K-FEV Scores from Pre- to Post-Intervention in Face-to-Face vs. Telehealth Cohorts. Bar graph illustrating mean change (Δ = Post − Pre) in K-FEV subscale scores—Cognitive Control, Disruptibility, and Hunger Sensations—after 12-month intervention in the matched face-to-face (dark grey) and telehealth (light grey) groups. Positive values indicate improvements in the respective domains. Error bars represent standard deviations.
• Physical performance (6MWT) remained improved.
• HrQoL scores and appetite regulation remained stable at elevated levels.

Longitudinal follow-up was conducted exclusively in the telehealth group. Sustained improvements were observed across all measured domains:

• BMI SDS reductions were maintained at 24 and 36 months (Fig. 5).

### 3.7 Safety and adherence

No adverse events were reported in either group. All participants completed the 12-month intervention, with the telehealth cohort also completing the follow-up. Protocol adherence was high in both cohorts, with 88.7 % in the telehealth group and 85.2 % in the face-to-face group. While this reflects slightly higher engagement in the telehealth model, the difference was not statistically significant.

## 4 Discussion

This retrospective cohort study assessed the long-term effectiveness of a structured, multimodal pediatric obesity intervention delivered via telehealth compared with a historical face-to-face cohort. Despite baseline disadvantages in the telehealth group—such as slightly elevated BMI SDS and reduced health-related quality of life (HrQoL)—the intervention demonstrated comparable, and in some domains superior, efficacy across clinical, behavioral, and psychosocial outcomes, with sustained effects observed up to 36 months.

The pandemic-induced transition to telehealth was not only a logistical response to public health restrictions but also a catalyst for innovation in chronic disease management. Children and adolescents during the COVID-19 pandemic experienced widespread disruptions in lifestyle routines, with studies reporting increased sedentary behavior, decreased physical activity, and a shift toward unhealthy dietary patterns, all of which contributed to rising BMI and declining psychosocial well-being [14,24,25]. In this context, our findings highlight the potential of telehealth to counteract such adverse trends through structured digital interventions.

### 4.1 Anthropometric and metabolic effects

Reductions in BMI SDS were observed in both delivery models, aligning with findings from prior pediatric obesity interventions [26,27]. Importantly, the telehealth cohort maintained BMI SDS reductions at both 24 and 36 months, underscoring the durability of intervention effects. While many obesity trials report high attrition and weight regain post-treatment [28], the sustainability observed here is notable and may be attributable to the continued family involvement and remote support.

The intervention also yielded favorable changes in insulin sensitivity, with a significant reduction in the HOMA-IR index. This is consistent with studies showing that modest weight loss and lifestyle modifications improve insulin dynamics in youth with obesity [29]. While reductions in lipid parameters (LDL-C, triglycerides) and HbA1c were not statistically significant, trends mirrored those reported in face-to-face interventions [30,31]. Liver enzyme levels (GOT) remained stable, indicating no hepatic compromise—an important consideration given the high prevalence of non-alcoholic fatty liver disease in pediatric obesity [32].

### 4.2 Physical fitness and body composition

Physical fitness, measured via the 6-min walk test (6MWT), improved significantly in both groups. These findings support prior evidence that home-based, age-appropriate activity regimens can effectively enhance aerobic capacity in pediatric populations [33]. While detailed body composition data (e.g., visceral fat, android fat) were not collected, improvements in performance suggest favorable changes in functional capacity and likely in lean body mass.

### 4.3 Behavioral and psychosocial outcomes

Improvements in dietary behavior were more pronounced in the telehealth cohort, with significantly increased consumption of vegetables, fruits, and reduced intake of unhealthy snacks. These findings parallel the work of Struckmeyer et al. [13], who observed substantial shifts in food intake patterns during structured remote interventions. The inclusion of parents in the education modules may have contributed to this outcome by reinforcing healthy norms at the household level [34].

Cognitive control over eating, as assessed by the validated Kinder-Eating Behavior Questionnaire (K-FEV), improved significantly in the telehealth group, with sustained gains at 24 and 36 months. This enhancement in appetite regulation is essential for long-term weight management and reflects the integration of behavioral therapy components into the intervention [35].

Most notably, HrQoL—often overlooked in obesity trials—improved across all domains, with lasting effects beyond the active intervention. The magnitude of improvement in the emotional well-being and family domains is particularly relevant, given the established bidirectional relationship between psychosocial stress and obesity-related behaviors [36]. These findings align with those of Reschke et al. [14], who reported comparable QoL improvements in a telehealth-based pediatric cohort during COVID-19 lockdowns.

### 4.4 Adherence and safety

The high protocol adherence (>85 %) and absence of adverse events affirm the acceptability and safety of the telehealth modality. These metrics are especially significant given that treatment adherence in pediatric obesity programs typically declines over time [37,38]. The digital format may have facilitated flexibility, reduced logistical burdens, and promoted engagement.

### 4.5 Telehealth as a component of multimodal care

While the telehealth format demonstrated clinical efficacy and sustainability, it is important to emphasize that structured digital interventions are not equivalent to passive recreational screen time. The therapeutic use of video platforms in this program was time-bound, interactive, and goal-oriented, thus clearly distinguished from sedentary screen exposure typically linked to obesity.

Furthermore, although our findings support the utility of a structured telehealth approach during pandemic conditions, we do not propose it as a universal replacement for face-to-face care. A hybrid model—combining digital delivery with intermittent in-person visits—may offer the most practical and effective strategy for long-term pediatric obesity care.

Finally, the observed improvements must be interpreted within the broader context of life circumstances, behavioral adaptations, and program adherence. While telehealth played a key role, causal attributions should be made with caution.

### 4.6 Limitations

This study has several limitations that should be acknowledged. First, the retrospective observational design introduces potential bias, despite the use of propensity score matching to control for baseline differences. Second, the use of historical controls may be confounded by secular trends, seasonal variation, or unmeasured pandemic-related influences. Third, data were obtained from a single center, which may limit the generalizability of findings to other settings or populations. Fourth, while validated instruments were employed for behavioral and psychosocial assessments, self-report bias cannot be excluded. Although propensity score matching was applied to reduce baseline confounding, key behavioral and psychosocial parameters such as dietary behavior, physical activity, and HrQoL were not included in the matching model. These variables differed at baseline and reflect contextual differences between cohorts, particularly the impact of the COVID-19 pandemic on the telehealth group. As such, full comparability cannot be assumed, and observed effects should be interpreted with caution regarding potential residual confounding. Participation in the telehealth cohort required access to stable internet connections and compatible digital devices, which may have inadvertently excluded families from lower socioeconomic backgrounds. This digital prerequisite introduces a potential selection bias, as these families may have been able to participate in the face-to-face cohort but were underrepresented in the telehealth group. Consequently, the observed outcomes may not be fully generalizable to all pediatric obesity populations, particularly those with limited digital access. Lastly, although adherence rates were high, we did not assess fidelity to individual components of the telehealth intervention, such as session content or family engagement intensity.

## 5 Conclusion

This retrospective observational study demonstrated that a structured telehealth-based lifestyle intervention for children and adolescents with obesity was effective in achieving significant improvements in BMI SDS, physical fitness, metabolic markers, dietary behavior, cognitive appetite regulation, and health-related quality of life. Importantly, these benefits were sustained over a 36-month period, indicating the durability of intervention effects. Compared to a historical face-to-face cohort, the telehealth model yielded comparable or superior outcomes across multiple domains, highlighting its clinical potential as an alternative or complementary modality in pediatric obesity care. These findings support telehealth as a clinically viable and sustainable model for pediatric obesity management, where even weight stabilization during linear growth may lead to improvements in BMI SDS and associated health outcomes.

## 6 Clinical relevance takeaways

• Telehealth interventions can deliver sustainable improvements in weight status, dietary habits, and psychosocial well-being in youth with obesity.
• Longitudinal data demonstrate that virtual care models maintain efficacy over three years, supporting their integration into chronic care strategies.
• Remote delivery is safe, feasible, and well-tolerated by families, offering a scalable solution to improve access to pediatric obesity care.

## Informed consent statement

The study was conducted in accordance with the Declaration of Helsinki. Written informed consent was obtained from all participants and their legal guardians. Participant confidentiality and data protection were ensured throughout the study.

## Ethics review statement

This study was a retrospective observational analysis based on anonymized routine clinical data obtained from a standardized outpatient obesity management program. No interventions beyond standard care were performed. In accordance with the institutional policy of the Hannover Medical School, ethical approval was not required for the secondary analysis of retrospective data. The study was conducted in association with the Institute for History, Ethics and Philosophy of Medicine at Hannover Medical School, adhering to the principles of the Declaration of Helsinki.

## Author contributions

N.S. and F.R. conceptualized and designed the study, developed the data collection tools, conducted the initial analyses, drafted the manuscript, and critically revised it. C.W., K.v.S., E.S., K.K., C.G., L.G., J.W., D.M., and N.S. were involved in data collection, while F.R. coordinated and supervised the data acquisition process. O.K., K.L., T.D., and T.B. provided critical revisions and contributed important intellectual input. All authors have read and approved the final version of the manuscript.

## Data availability statement

The data presented in this study are part of the KiCK program and were collected and analyzed at the Center for Clinical Studies, Children's Hospital AUF DER BULT, Hanover, in collaboration with the Department of Medical Psychology, Hanover Medical School. Source data are available upon request.

## Clinical trial registration

Not applicable. This was a retrospective observational study using historical clinical data and therefore not subject to prospective clinical trial registration requirements.

## Use of artificial intelligence

In this study, no artificial intelligence (AI) or machine learning algorithms were utilized for data collection, analysis, or interpretation. All data analyses were conducted using conventional statistical methods, and intervention delivery was performed through standard telehealth and face-to-face protocols without AI-assisted decision-making. Future studies may explore the integration of AI tools to enhance individualized treatment planning and predictive analytics in pediatric obesity management.

## Funding

This research was supported by the Dr. August und Erika Appenrodt Foundation (Grant ZE AAS-134).

## Declaration of competing interest

The authors declare that they have no known competing financial interests or personal relationships that could have appeared to influence the work reported in this paper.
