# Supplementary material for: Evaluating the long-term effectiveness of a structured telehealth obesity program in children and adolescents: A retrospective matched-control study
Source: Obes Pillars. 2025 Aug 29;16:100206. doi: 10.1016/j.obpill.2025.100206 (PMC12445613; doi:10.1016/j.obpill.2025.100206)
Supplement: Multimedia component 1 [file mmc1.docx]

# Supplementary Table S1. Dietary Consumption Frequencies by Food Group and Intervention Mode

Values represent the number and percentage of participants reporting a healthy or unhealthy frequency of consumption for each food group, categorized according to predefined criteria (≥4 servings/week = healthy for healthy foods; ≤3 servings/week = healthy for unhealthy foods). Statistical significance refers to between-group comparisons.

| Food Item | Face-to-Face Healthy n (%) | Face-to-Face Unhealthy n (%) | Telehealth Healthy n (%) | Telehealth Unhealthy n (%) | p Healthy | p Unhealthy |
| --- | --- | --- | --- | --- | --- | --- |
| Soft drinks | 60 (50.3%) | 59 (49.7%) | 40 (34.5%) | 77 (65.5%) | < 0.05 | < 0.001 |
| Mineral water, tea, water | 83 (69.2%) | 37 (30.8%) | 59 (50.4%) | 58 (49.6%) | < 0.05 | < 0.001 |
| Milk, Cocoa | 67 (55.9%) | 53 (44.1%) | 56 (48.4%) | 61 (51.6%) | n.s. | < 0.05 |
| Yoghurt, buttermilk, curd | 58 (48.5%) | 62 (51.5%) | 59 (50.3%) | 58 (49.7%) | n.s. | n.s. |
| Cheese | 54 (45.3%) | 65 (54.7%) | 70 (59.8%) | 47 (40.2%) | < 0.05 | < 0.05 |
| Meat/sausages | 33 (27.8%) | 86 (72.2%) | 15 (12.8%) | 102 (87.2%) | < 0.05 | < 0.01 |
| Fish | 10 (8.7%) | 110 (91.3%) | 4 (3.7%) | 113 (96.3%) | < 0.05 | n.s. |
| Mixed bar, white bread, rolls | 57 (47.4%) | 63 (52.6%) | 68 (58.4%) | 49 (41.6%) | n.s. | n.s. |
| Wholemeal bread, wholemeal rolls | 33 (27.8%) | 87 (72.2%) | 26 (23.5%) | 84 (76.5%) | n.s. | n.s. |
| Rice/Pasta | 34 (28.7%) | 86 (72.3%) | 12 (10.7%) | 100 (89.3%) | n.s. | < 0.05 |
| Fresh fruit | 46 (37.9%) | 74 (62.1%) | 73 (62.9%) | 43 (37.1%) | < 0.05 | < 0.05 |
| Vegetables (fresh/frozen) | 44 (36.5%) | 76 (63.5%) | 12 (10.5%) | 105 (89.5%) | < 0.05 | < 0.01 |
| Fastfood/Delivery Food | 38 (31.2%) | 84 (68.8%) | 5 (4.7%) | 102 (95.3%) | < 0.05 | < 0.01 |
| Chocolate/Sweets | 27 (22.9%) | 91 (77.1%) | 9 (7.8%) | 108 (92.2%) | < 0.05 | < 0.001 |
| Chips, salty snacks | 35 (28.9%) | 86 (71.1%) | 5 (4.2%) | 112 (95.8%) | < 0.05 | < 0.01 |
| Gummi bears, wine gum | 45 (37.8%) | 74 (62.2%) | 16 (13.8%) | 101 (86.2%) | < 0.05 | < 0.01 |
| Cakes, tartes, biscuits | 66 (54.7%) | 54 (45.3%) | 92 (78.9%) | 25 (21.1%) | < 0.05 | < 0.001 |
